# The Enduring Health Challenges of Afghan Immigrants and Refugees in Iran: A Systematic Review

**DOI:** 10.1371/currents.dis.449b4c549951e359363a90a7f4cf8fc4

**Published:** 2017-07-21

**Authors:** Nasim Sadat Hosseini Divkolaye, Frederick M. Burkle

**Affiliations:** International Affairs Department, Iranian Blood Transfusion Organization, Tehran, Iran; Harvard Humanitarian Initiative, Harvard University, Cambridge, Massachusetts, USA; The Woodrow Wilson International Center for Scholars, Washington, DC, USA

**Keywords:** Afghan immigrants, Afghan refugees, communicable diseases, food security, Global health, Iran health system, Non-communicable diseases

## Abstract

**Introduction:**

Iran is the third country in the world with the highest number of registered refugees with the majority coming from Afghanistan. They suffer major health and social risks yet their health status has never been comprehensively determined.

**Methods:**

This systematic review of the literature highlights major disparities among documented immigrants in health access, communicable and non-communicable diseases and the increasingly desperate plight of undocumented immigrants.

**Results:**

Comparing with Iranian population, the findings suggest the higher prevalence of most diseases among Afghan immigrants and refugees. This highlights the importance of increasing the migrants' access to health services from both public health as well as human rights perspectives.

**Discussion:**

Although the Iranian government has taken new initiatives to overcome this challenge, certain issues have still remained unaddressed. Potential solutions to improve this process are discussed.

## INTRODUCTION

One of the effects of globalized world is the increase of human mobility across the borders resulting in rapid growth of international migration. According to the 2015 United Nations International Migration report the number of international migrants has increased significantly during the past fifteen years reaching 244 million in 2015, up from 173 million in 2000^1^.

The huge number of displaced populations has turned migrant health into a priority global health priority. Although international migration may have some benefits, immigrants are usually among the most vulnerable groups in destination countries. Migrants are commonly subjected to multiple discriminations, violence or exploitation which may have considerable impact on their mental and physical health. According to a report of World Health Organization (WHO), in some countries migrants find themselves completely excluded from routine health services.^2^

Immigrants and refugees are at higher risk of developing certain diseases. Migrants originating from areas of poverty or those who are displaced by conflict or natural disaster are at greater risk of adverse health outcomes.^3^ In a study conducted by The Tuberculosis (TB) and Human Rights Task Force, refugees have a high risk of developing TB associated with poor nutritional status and sanitation, crowded living conditions, insufficient access to care, education and information, and other coexistent illnesses.^4^ Although it has always been a controversial issue for the host country, addressing the health needs of immigrants and refugees can improve health status and outcomes; facilitate integration; prevent long-term health and social costs; contribute to social and economic development; and, most importantly, protect public health and human rights.^5^

Iran is the third country in the world with the highest number of registered refugees (1 million).^1^ The majority of refugees came from Afghanistan but their health status has never been comprehensively determined. UNHCR acknowledges that “refugee” or “migrant” have distinct and different meanings and “confusing them leads to problems for both populations”. They use “refugees” when people flee war or persecution across an international border and “migrants” when people move for reasons not included in the legal definition of a refugee.^6^ In compliance, this study refers to Afghan refugees** as **nationals of Afghanistan who left their country as a result of war or persecution; and, Afghan immigrants as those who choose to move not because of a direct threat of persecution or death, but mainly to improve their lives by finding work, or in some cases for education, family reunion, or other reasons.^6 ^These terms are used interchangeably in some of the reviewed literature which ineluctably has been reflected in this manuscript. Evaluating the health needs of this population and assess their access to health services are necessary for health policymakers to develop and adopt appropriate strategies. Increasingly, this has become a major public health concern. As such, a systematic review of relevant studies including the culture profile, and health access and risks are required to better assess and respond to issues of prevention, preparedness, response and recovery.

**METHODS**

All national (MagIran, Science Information Database (SID) and Iranmedex) and international (PubMed, Scopus) databases were searched from November 2010 to November 2016 using keywords both in English and Persian: Afghan immigrants, Afghan refugees, Iran, infectious diseases, tuberculosis, HIV, Hepatitis B and C, non-communicable disease, food security, mental health, barriers, health insurance, access to health service. All related websites and webpages were also searched by Google with the same keywords. The author also used back-tracking to find earlier relevant sources from 2001.

This literature review resulted in 86 articles. This process preferenced systematic reviews but due to small sample sizes of cases studied additional cases where found in humanitarian organizational reports and webpages. The final number of articles included: 8 systematic reports, 24 original articles, 7 organizational and 5 webpages.

**RESULTS**

**Cultural Profile of Afghan Immigrants**

Following the political disruptions in Afghanistan, the Islamic Republics of Iran and Pakistan experienced a massive influx of Afghan refugees during the past three decades. Currently, more than 2.5 million Afghan immigrants live in Iran accounting for 3% of total Iranian population.^1^ the data of this study is primarily based on the health status of documented refugees in Iran and does not contain the situation of more than 1.5 million illegal immigrants.

Over the past decade under the so-called Amayesh record system,^7^ the Iranian authorities have only allowed Afghans who arrived before 2001 and those who have been in Iran for a long time to register in the system and obtain legal residence. Afghans who have arrived after 2001 are now considered illegal immigrants.^8^ The latest registration (Amayesh XI) was completed in 2016.^7^

Ninety-seven percent of Afghan refugees live in urban areas while 3% reside in settlements and camps run by the assistance of the government, UNHCR and foreign NGOs.^9^ The Afghan immigrant population is relatively young in Iran with a median age of 31 years. In compliance with the world trend, in 2015 less than half of the international immigrants in Iran were women (47%).^1^ One-third of immigrants (32.7%) resided in Tehran, 13.3% in Khorasan Razavi (Mashhad), 11.7% in Isfahan, 9.3% in Sistan & Baluchistan, and the remainder in other provinces.^10^

A survey performed among registered Afghan employees in 2006, found that low educational attainments characterized the surveyed Afghan population. Thirty-one per cent of the population aged six and above in this sample were uneducated (women 36%, men 26%) and 50% had completed only primary or secondary school education. The average household size of Afghan population in Iran is 5.6 persons. About 80% of Afghans work in four sectors - manufacturing, construction, trade, and commerce. Less than 3% of the Afghan employees had written contracts and more than 99% of Afghan employees did not have any type of work-related insurance (accident, unemployment and retirement insurance) and only 5% were entitled to paid annual or sick leave. The majority of households (83%) live in rented houses. The main reason for their immigration was escaping from war and insecurity.^11^

In 2013-2014, more than 350,000 Afghan refugee children were registered in Iranian schools,^9^ while some 48,000 undocumented Afghan children were allowed to enroll for the first time in Iranian public schools in 2015.^12^

**Health Problems of Afghan Immigrants**

The health needs of Afghan immigrants and refugees in Iran are quite similar to other immigrants around the world. Although the lack of data is much more visible in some diseases, this study has attempted to provide a general overview to the most important health needs of Afghan immigrants and refugees in Iran. The main health problems of Afghan immigrants/refugees have been categorized into three sections: mental, communicable and non-communicable diseases.

**Mental Health**

In a systematic review of multiple countries on the long-term mental health of war affected refugees, the prevalence rate of depression ranged between 2.3 to 80%, posttraumatic stress disorder (4.4–86 %), and unspecified anxiety disorder (20.3–88 %). This heterogeneity in prevalence rates was mostly contributed to the methodological quality and which country the refugees came from and in which country they resettled.^13^

Some studies identified the prevalence of mental disorders among Afghan refugees in different parts of Iran. In a study designed to determine the prevalence of mental health problems among Afghan refugees resettled in Dalakee refugee camp of Bushehr Province, the prevalence of social dysfunction, psychosomatic problem, anxiety and depression in the studied population were 80.1%, 48.9%, 39.3% and 22.1%, respectively. In this study, the total prevalence of mental health disorders was determined as 88.5%.^14^ Also findings of another study conducted in Tehran showed that the prevalence of mental disorders was 55.6% (19.9% in males and 35.7% in females) among Afghan immigrants.^15^

Compared to the studies conducted among Iranian population,^16^ the prevalence of mental disorders is relatively higher among Afghan refugees especially those living in settlements and camps.

**Communicable Diseases**

Several studies report a relative high prevalence of Malaria,^17^ Hepatitis B,^18^ Tuberculosis,^19^ Cholera,^20 ^Crimean–Congo hemorrhagic fever,^21^ leishmaniasis,^22^ and HIV among Afghan immigrants in Iran.^23^

Tuberculosis, MDR tuberculosis and malaria are the most common infectious diseases among the Afghan immigrants in Iran. In a systematic review and meta-analysis study done on major infectious diseases affecting Afghan population in Iran, the proportion of Afghan immigrants who were infected with tuber­culosis was (29%), Multiple-Drug-Resistant (MDR) tuberculosis (56%), malaria (40%), cholera (8%), Crimean-Congo hemorrhagic fever (25%), leishmaniasis (7%), and hepatitis B (14%). The overall proportion of Afghan immigrants with the aforementioned infectious diseases is 29%.^24 ^In 2008, Jabarii and colleagues assessed the prevalence of HIV among Afghan immigrants to be 0.2% in a town to the northeast of Tehran.^23^

There is a huge difference between the prevalence of infectious diseases among Afghan immigrants and Iranian population. While the prevalence of some infectious disease such as tuberculosis and malaria is high among Afghan immigrants, Iran has almost eradicated both diseases among its nationals.^25^^,^^26^ Also, evidence suggests that the prevalence of hepatitis B is estimated to be about 1.7% among Iranians^27^ which is significantly lower than the Afghan immigrant population.

**Non-communicable disease**

Non-communicable diseases (NCDs) represent 43% of the burden of disease worldwide. The Middle East is known to have high rates of major NCDs such as heart disease, stroke, cancer, diabetes and chronic lung disease with risk factors that are the main causes of morbidity and mortality.^28^ The current data from Afghanistan show that the rate of NCDs is increasing with more than 35% mortality.^29^

According to data extracted from 23,167 registered Afghan refugees who were referred to United Nations High Commissioner for Refugees (UNHCR) offices from 2005 to 2010, the most common health referral for females and males (0–14 years) was perinatal diseases. In the females aged 15 to 59 it was ophthalmic diseases (13.65%), and for males it was nephropathies (21.4%). Overall, in both sexes the most frequent causes for referrals were for ophthalmic diseases, primarily cataracts (23.7%), neoplasm (13.3%), nephropathies (11%), ischemic heart diseases (10.4%), and perinatal disorders (9.2%).^30^

In 2011, a study was carried out to compare the prevalence of premature newborns’ birth among Afghan and Iranian ethnics. The rate of preterm birth prevalence was 7.1% (391 cases) and 14.5% (56 cases) among Iranian and Afghan populations respectively. Low birth weight prevalence was 6.7% (367 cases) among Iranians and 12.7% (49 cases) among Afghans. The study also found that preterm birth complications are almost two times more among Afghan immigrants than Iranians.^31^

A study was designed to evaluate the prevalence of food insecurity and its socio-demographic determinants among Afghan immigrants in two major cities of Iran. The results indicated that more than 60% suffered from moderate-to-severe food insecurity, 14% were mildly food-insecure while about 23% were food-secure. Food insecurity was significantly more prevalent in female-headed households, those with illegal residential status, unemployment/low job status, not owning their own home and low socioeconomic status.^32^ The prevalence of food insecurity among Iranians was reported in 2015 in a meta-analysis as 49% among households, 67% in children and 61% in mothers.^33^

**Access to Health Services**

Refugees have special health needs. Their fragile situation which arises from the experiences they had in their homeland and difficulties they may encounter in the host country put them at risk for developing mental and physical disorders. Improving the access to health services of this population not only is an essential human right but also has major benefits for the population as a whole.

There is scant of evidence in Iran regarding the use of health care services by Afghan immigrants and asylum seekers. According to a UNHCR report, during the past three decades, Afghan refugees have had access to basic health care, education, and employment opportunities. However, the financial constraints and lack of international support has always been a main barrier for the government to comprehensively take necessary actions. In 2014, through a joint collaboration of a private insurance company, UNHCR and Ministry of Interior, more than 220,000 vulnerable Afghan refugees including 2000 refugees with special diseases (Hemophilia, Thalassemia, Dialysis, Kidney Transplant and Multiple Sclerosis) were provided insurance services. The Government and UNHCR also provided primary health care in 15 settlements, camps and 29 urban locations.^8^

In addition, since 2016, Iran has started to enroll all registered Afghan refugees (more than 950,000) under Public Health Insurance. The refugees will benefit from a health insurance package for hospitalization identical to the scheme available to Iranian nationals. The insurance covers entire treatment expenses for people with special diseases and vulnerable groups (families who have patients with incurable disease or mental/physical disabilities, children of Iranian widows who married Afghan nationals, female-headed households, families who have nine or more children, poor people, the households whose their head is not able to work due to the medical conditions or disability, Afghan nationals who married Iranian women, the head of households with 65 or more years old, unmarried men and women with more than 75 and 18 years old).^8^ As of October 2016, more than 250,000 foreign nationals have been covered under this insurance. The initiative took place when a tripartite memorandum of understanding was signed between Ministry of Labor, Ministry of Health and Ministry of Interior in 2015. The beneficiaries of this scheme provide a contribution to the funding; however the Government of Iran covers half of the real monthly costs of the insurance premium. This is further complemented by a UNHCR contribution of 8.3 million USD for this six month period by primarily focusing on vulnerable refugees.^34^

In 2008, the Executive Director of the United Nations Office on Drugs and Crime (UNODC) and the Deputy Secretary General of the Islamic Republic of Iran's Drug Control Headquarters signed an agreement to provide HIV prevention and care services to Afghan refugees and female drug users in Iran. These services were launched through funding from the Government of the Netherlands.^35^

However, the situation of unregistered Afghans remains unclear. Basically, undocumented Afghans cannot register for health insurance and therefore have limited access to the public health service. According to an independent body’s report unregistered Afghans are able to obtain treatment at private health institutions, but they must pay for the treatment from private funds. They can also benefit from the free health services provided by some NGOs and charities or on an individual basis.^8^

**Barriers to Access to Health Services**

The illegal status of almost 1.5 million Afghan immigrants prevents them to access to health insurance and consequently limits their access to health services. This situation gets worse considering a large number of illegal afghan immigrants work in hard and hazardous jobs such as the construction sector^36^ where the risk of injuries is relatively high. Additionally, more than 99% of Afghan employees do not have any type of work-related insurance.^11^

In a literature-review study conducted in 2015, the authors categorized the barriers to health care for undocumented immigrants in three levels: the problems that exist in laws and policies of destination countries including limitations to access and type of health care, the barriers within health system that included bureaucratic obstacles including paperwork and registration systems and finally the hindrance that exists at the individual level focused on the immigrant’s fear of deportation, stigma, and lack of capital (both social and financial) to obtain services.^37^

A large number of Afghan refugees and immigrants in need of health care in Iran are among poor and economically vulnerable groups. Many refugees and immigrants struggle to find work and often take jobs with low wages. According to one survey, Iranian workers benefit from 10-23% higher wages compared to Afghans.^1^ ^9^ This inability to pay and lack of a comprehensive health insurance have led to the late self-referral of immigrants/refugees to health care services when the disease is in advanced stages. In one study completed among Afghan refugees to detect their common kidney diseases, it was found that due to the cost of medical visits or medications, the most common health referral for Afghans was end-stage renal disease (ESRD).^38^ Language barriers and lack of communication are mentioned by several studies as the main obstacles to refugee health care access worldwide, is not the case for Afghan immigrants in Iran.^39^^,^^40^

**LIMITATIONS**

Although the governmental institutions are able to provide reliable data on the situation of Afghan immigrants/refugees and conduct extensive research, the data used in this study is driven from independent researches, and reports of international organizations and foreign NGOs.

**DISCUSSION**

Providing health care for immigrants/refugees is crucial from two different aspects: First, immigrants may increase the potential risk of spreading some communicable diseases among the national population. Several studies indicate that the Afghan immigrants have contributed to the spread of communicable diseases in Iran with an estimated 55% of new multi-drug resistant tuberculosis patients, 40% of malaria patients, 29% of tuberculosis patients and 25% of Crimean-Congo hemorrhagic fever patients in Iran are Afghan immigrants respectively.^23^ Illegal migration poses a serious threat to the disease elimination program of Malaria in Iran.^39^ It has been estimated that the government spends more than 100,000 USD annually just to treat Afghan immigrants with tuberculosis.^40^

The second concern is related to human rights which put the emphasis on adequate and equitable access of immigrants to health services. The right of everyone to the enjoyment of the highest attainable standard of physical and mental health has long been established in international human rights law such as the International Convention on the Elimination of All Forms of Racial Discrimination (ICERD) and the International Covenant on Economic, Social and Cultural Rights.

Unfortunately, there is a gap between evidence and policies in Iran. The government has not yet formulated a comprehensive policy to address the different health risks and needs of the immigrants. Considering the health and financial burden of immigrants on the host country, exclusion of immigrants from health services is not a wise approach both in terms of public health as well as human rights. Although the current initiative of the government to provide health insurance for registered Afghans was a big step forward, the plan has some major deficiencies. Firstly, the way to deal with the health needs of 1.5 million unregistered Afghan is still under question. This gains importance knowing that immigrants whose legal situations has not yet determined are significantly at higher risks of contracting disease and in developing mental health problems due to their living situations.^41^ Secondly, the plan entails the financial support of external donors which has always been a controversial issue.

As a part of the implemented policies, the Iranian government has put much attention on repatriation policies. From 2002 to 2014, the number of Afghan refugees who returned to their homeland voluntarily was 920,161.^9^ However, repatriation should not be considered as a single policy. Given the complex process of migration and its health consideration at multiple phases (pre-departure, travel, Destination, Interception and return phases),^42^ dealing with this problem needs a long-term, multi-sectorial approach (collaboration between government, intergovernmental organizations and civil society).

Currently by the joint initiative of Afghanistan, Iran, Pakistan and UNHCR, a Solution Strategy for Afghan Refugees (SSAR) was developed to find and implement a comprehensive solution for Afghan refugees in the region. The SSAR also seeks to improve access to health services and support from the Iranian government to this end by contribution of several partners such as governmental and international organizations, NGOs and civil society.^9^ Failing to address to the situation of undocumented immigrants, SSAR encompasses the same flaws as government’s insurance plan. In addition, the health solution strategies are relatively scant compared to those that addressed the education and skill training of refugees.

Given the significant threats posed by limits on illegal immigrants’ access to Iranian health system, formulation of a comprehensive and uniform strategy addressing health care needs of illegal immigrants is necessary. The current approach of the government is ignoring the problem of huge number of illegal immigrants which as stated above is not a wise approach.

The government should be persuaded to change its current legislation on illegal migration. As a part of this policy, it is recommended to extend the time needed for accepting the legal status of refugees (currently the refugees who came before 2001 are allowed to apply for legal authorization). In this context, the existence of strong civil society and NGOs to push the government to change its approach is crucial.

## Conflict of Interest

The authors declare no conflict of interests.

## Funding

The authors received no specific funding for this study.

## Ethics Statement

Ethical approval: This article does not contain any studies with human participants or animals performed by the author.

## Data Availability

All national (MagIran, Science Information Database (SID) and Iranmedex) and international (PubMed, Scopus) databases were searched from November 2010 to November 2016 using keywords both in English and Persian: Afghan immigrants, Afghan refugees, Iran, infectious diseases, tuberculosis, HIV, Hepatitis B and C, non-communicable disease, food security, mental health, barriers, health insurance, access to health service. All related websites and webpages were also searched by Google with the same keywords and used back-tracking to find earlier relevant sources from 2001.

## Corresponding Author

Frederick M. Burkle, Jr. Email: burkle@hsph.harvard.edu Tel: 1-808-262-2098 (Hawaii)
